# Supplementary material for: Content-rich biological network constructed by mining PubMed abstracts
Source: BMC Bioinformatics. 2004 Oct 8;5:147. doi: 10.1186/1471-2105-5-147 (PMC528731; doi:10.1186/1471-2105-5-147)
Supplement: Additional File 2 — The original results of the above study (non-essential files are deleted to keep the file size under the limit set by BMC bioinformatics). [file 1471-2105-5-147-S2.bz2 › chilibotAdditionalFile2/dip20/81ID8798570E293/html/EPHB1_GRB2.html]

 


 **EPHB1** and **GRB2** 
  
Found 15 abstracts in PubMed, retrieved 15.  
 

 What does Google say? 
 PDF only 
| .edu only 

---

**Interactive relationship** (e.g. stimulation, inhibition, etc)

**Neutral relationship**- These findings define ligand activated interaction between ELK  [ **EPHB1** ]  and the SH2 domains of  **Grb2**  and the newly identified Grb10 protein that shares homology with a Caenorhabditis elegans gene product implicated in neural cell migration.  Ref: 8798570 J Biol Chem, 1996
- Ligand activation of ELK  [ **EPHB1** ]  receptor tyrosine kinase promotes its association with Grb10 and  **Grb2**  in vascular endothelial cells.  Ref: 8798570 J Biol Chem, 1996
- Both the catalytic activity and, to a much lesser extent, the  **Grb2**  binding tyrosyl phosphorylation sites of SHP 2 are required for maximal FGF 2 induced Erk activity and Elk  [ **EPHB1** ]  1 transactivation.  Ref: 11997521 Mol Cell Biol, 2002

**Non-interactive relationship** (e.g. studied together, co-existance, homology, etc.)

- The ELK  [ **EPHB1** ]  ligand, LERK 2 Fc, stimulated tyrosine phosphorylation of ELK  [ **EPHB1** ] , and recruitment of Grb10 and  **Grb2**  to endothelial ELK  [ **EPHB1** ]  receptors recovered by wheat germ agglutinin lectin and immunoprecipitation.  Ref: 8798570 J Biol Chem, 1996
